# Eight weeks of post‐exercise local heating does not improve cognition and plasma brain‐derived neurotrophic factor concentrations

**DOI:** 10.1113/EP092810

**Published:** 2025-12-13

**Authors:** Jem L. Cheng, Geoff B. Coombs, Christina Pizzola, Keira Mattook, Calvin Armstrong, Maureen J. MacDonald, Jeremy J. Walsh

**Affiliations:** ^1^ Department of Kinesiology, Faculty of Science McMaster University Hamilton Ontario Canada; ^2^ Institute for Applied Human Physiology, College of Medicine and Health Bangor University Wales UK

**Keywords:** brain‐derived neurotrophic factor, cognitive function, exercise training, hot water immersion, post‐exercise heating

## Abstract

Exercise and heat stress have been reported to independently provide benefits to brain health. We tested the hypothesis that 8 weeks of post‐exercise local heating, passive local heating only, or exercise training only improves cognitive performance compared to a control group. Sixty young, healthy participants (*n* = 30 female, age: 23 [3] years) were randomised into one of four groups: control (CON), aerobic exercise (EX), local heating (HEAT), or combined heat and exercise (HEATEX). Participants completed supervised sessions three times per week for 8 weeks. Exercise sessions were completed at 70–75% of maximum heart rate on a cycle ergometer, and local heating sessions involved hot water immersion (42°C) of the feet (both 45 min duration). The HEATEX group performed both the EX and HEAT components sequentially in the same session (90 min total duration). Cognitive performance was measured at baseline and at the end of the 8‐week intervention using the digit symbol substitution task (DSST) and the Stroop test. There was a main effect of time (*P *< 0.001) where DSST performance improved; however, there was no group effect (*P *= 0.089) or time by group interaction (*P *= 0.119). There was no effect of the interventions on Stroop cost (baseline: 90 [SD: 70] ms; post‐intervention: 84 [SD: 70] ms; time by condition interaction *P *= 0.205). Similarly, there were no effects of the interventions on circulating plasma concentrations of brain‐derived neurotrophic factor (interaction *P *= 0.189). Eight weeks of exercise training and/or local heating is not sufficient to improve cognitive performance in young, moderately fit individuals.

## Abstract (highlights)

- **What is the central question of this study?**
  What are the effects of 8 weeks of exercise training, post‐exercise local heating, and a combination of the two on cognition and circulating BDNF?
- **What is the main finding and its importance?**
  There were no benefits to cognitive performance, including reaction time or response accuracy, nor was circulating BDNF changed following 8 weeks of aerobic training, lower limb heating, or a combination of exercise and heat.

## 1 INTRODUCTION

Heat therapy, broadly defined as regular exposure to any form of passive heating stimulus, confers many systemic physiological benefits to humans (Cheng & MacDonald, 2019). Heat therapy has been reported to improve cardiovascular function (Brunt et al., 2016), reduce the risk of cardiovascular diseases (Laukkanen et al., 2015) and dementia (Laukkanen et al., 2017), and enhance exercise performance (Lorenzo et al., 2010). Indeed, acute heat exposure imposes large cardiovascular and autonomic stresses that require a highly integrative, whole‐body response. In this regard, the mechanisms underlying the health benefits of heat therapy have garnered consideration as a potential lifestyle intervention. The mechanisms underlying physiological adjustments to passive heating include vascular shear stress, upregulation of heat shock proteins and potentially other neurotrophic factors (Brunt & Minson, 2021). Although current evidence is sparse, the combination of various physiological stressors (e.g., heat and hypoxia or heat and exercise) may augment cellular adaptation and function via cross‐acclimation (Ely et al., 2014; Rodrigues et al., 2025; Stevens et al., 2025). With repeated exposures, these mechanisms can elicit adaptations that positively impact the physiological function of numerous systems, including the brain.

Repeated exposure to passive heating confers numerous physiological benefits, and epidemiological data indicate that regular passive heating is beneficial for brain health. More frequent and longer‐duration sauna bathing is associated with lower risk of mental health disorders (Laukkanen et al., 2018), stroke (Kunutsor et al., 2018) and dementias (Knekt et al., 2020; Laukkanen et al., 2017). However, experimental data on the cognitive responses to heat stress are limited. Evidence exists to demonstrate that acute whole‐body passive heating improves reaction time, but not accuracy, in younger and older adults (Schlader et al., 2015). However, the influence of repeated exposures to passive heating on brain health is poorly understood and the potential combined effects of exercise training and passive heating have yet to be explored. Post‐exercise heating via sauna or hot water immersion can induce heat acclimation responses characterised by improved thermoregulatory and cardiovascular responses (Zurawlew et al., 2019, 2016) that sustain elevated core temperature for longer durations. Similarly, several studies have demonstrated improved vascular function following localised heating to a particular limb (e.g., the lower leg or forearm) (Cheng et al., 2019; Tinken et al., 2009).

A convergent mechanism underlying the putative cognitive‐enhancing benefits of exercise or passive heat exposure may be the upregulation of brain‐derived neurotrophic factor (BDNF). BDNF is a neurotrophin that plays a critical role in orchestrating activity‐dependent neuronal and cognitive adaptations to exercise (Intlekofer et al., 2013). Emerging evidence demonstrates that acute whole‐body heating increases concentrations of serum and plasma BDNF (Kojima et al., 2018; Ogawa et al., 2021), suggesting that heat therapy may represent a strategy to increase circulating BDNF, thereby mirroring the capacity of aerobic exercise to potentiate cognition (Dinoff et al., 2017; Matthews et al., 2009). Indeed, it has been reported that repeated heat exposures increase circulating BDNF after 4 weeks (Glazachev et al., 2020). Mechanistically, exercise and passive heating both increase vascular shear stress, which stimulates the production and release of BDNF from vascular endothelial cells into the blood (Meuchel et al., 2011; Prigent‐Tessier et al., 2013). It is unclear whether local heating increases plasma BDNF concentrations, but previous work from our group indicates large increases in vascular shear rate in the brachial artery (+400%), thus supporting the putative mechanisms for greater circulating BDNF with foot heating (Cheng et al., 2021). Further, both stimuli increase sympathetic nervous system activity (Fujimura et al., 2002), which increases serum BDNF via platelets released from the spleen (thrombocytosis) (Bakovic et al., 2013). Taken together, the integrated physiological responses common to both exercise and passive heating may confer neural benefits resulting in improved cognitive function, possibly via increased BDNF concentrations in the blood. Whether these physiological responses are augmented by the combination of stressors via post‐exercise local heating has not been tested in this context.

The aim of this study was to investigate the effects of 8 weeks of either exercise training, or local heat therapy, or a combination of both on aspects of cognitive function. We tested the primary hypothesis that 8 weeks of a combined intervention of local heating and exercise improves cognitive performance compared to either intervention alone or a control group. We also tested the secondary hypothesis that combined exercise training and heat therapy elevates circulating BDNF to a greater extent compared to either modality alone. The addition of localised protocols can be more affordable and accessible for various populations without access to sauna/hot tubs (Romero et al., 2016; Thomas et al., 2016), or in clinical conditions that impair thermoregulatory capacity (Balmain et al., 2016; Coombs et al., 2018). Whether this type of combined intervention might also improve cognitive function due to increased haemodynamic stress or neurotrophic factors has not been previously studied.

## 2 METHODS

### 2.1 Ethical approval

This study reports secondary outcomes from a randomised controlled trial that investigated the effect of exercise training and local heat therapy on peripheral vascular function in young adults. Detailed information about the trial is reported elsewhere (Cheng et al., 2025). The trial was approved by the Hamilton Integrated Research Ethics Board (project no. 12723) and all participants signed written, informed consent prior to participation. This study conformed to the latest standards set forth in the *Declaration of Helsinki* and was pre‐registered with ClinicalTrials.gov (identifier no. NCT04588103).

### 2.2 Participants (Table 1)

We recruited young, healthy, recreationally active individuals between the ages of 18–35 years old who were cleared to participate in physical activity according to the Physical Activity Readiness Questionnaire (2020 PAR‐Q+). Exclusion criteria included smoking and/or vasoactive or recreational drug use; a history of cardiovascular, metabolic or musculoskeletal disease; and a maximal oxygen uptake (${\dot{V}}_{O_{2}\mathrm{peak}}$) ≥ 60 mL/kg/min at baseline.

### 2.3 Study design

Participants were randomised to either no intervention (CON), heat therapy (HEAT), aerobic exercise training (EX), or combined exercise training and heat therapy (HEATEX) conditions. The intervention period was 8 weeks in duration, and participants randomised to intervention conditions (i.e., HEAT, EX, and HEATEX) attended the laboratory three times per week to complete their assigned interventions in a supervised manner. Participants assigned to CON were asked to maintain their current physical activity and dietary habits and were only contacted to arrange their experimental visits. Outcome measures were assessed pre‐ and post‐intervention, and within 48–96 h following the final training session for those in intervention groups (48 h: *n* = 4, 72 h: *n* = 32, 96 h: *n* = 24).

### 2.4 Training interventions

#### 2.4.1 Heat therapy (HEAT)

Heat therapy involved 45 min of dual lower‐limb hot water immersion at 42.8°C (109°F) using a commercially available foot bath (IVATION, Edison, NJ, USA) filled to the ‘MAX’ line. For all participants, the water level was above the lateral malleoli.

#### 2.4.2 Exercise training (EX)

Exercise training involved 45 min of cycling at a moderate intensity corresponding with 70–75% of a participant's maximum heart rate (HR_max_; determined during ${\dot{V}}_{O_{2}\mathrm{peak}}$ test) on a stationary cycle ergometer (Lode Corival; Gronigen, The Netherlands/Kettler Ergorace; Ense, Germany/SCIFIT upright bike; Tulsa, OK, USA/LifeFitness 95 Ci; Toronto, ON, Canada). Each session began with a 3‐min warm‐up at 50 W, followed by 40 min of cycling at the prescribed intensity, and ended with a 2‐min cool‐down at 50 W.

#### 2.4.3 Combined training and heat therapy (HEATEX)

Combined exercise training and heat therapy involved 90 min of the EX protocol followed immediately by the HEAT protocol.

### 2.5 Experimental protocol

For all experimental visits, participants came to the laboratory having abstained from food, alcohol and caffeine and moderate to vigorous exercise for 6, 12 and 24 h, respectively. Upon arrival, participants rested in the supine position for 10 min whilst they were instrumented for measurements. A trained phlebotomist performed a blood draw before the assessment of resting cardiovascular haemodynamics. Participants performed a ${\dot{V}}_{O_{2}\mathrm{peak}}$ test to quantify cardiorespiratory fitness after all other measurements were taken. In separate visits to the laboratory pre‐ and post‐intervention, participants performed a cognitive test battery seated on a computer following a period of supine rest to quantify cognitive function.

### 2.6 Measurements

#### 2.6.1 Resting cardiovascular haemodynamics

Resting heart rate (HR) and blood pressure (BP) were measured in triplicate with an automated sphygmomanometer (Dinamap Carescape, V100; GE Healthcare; Mississauga, ON, Canada). Participants were in the supine position and asked to remain still and quiet for the duration of the assessment. The average of the second and third measurements was used for analysis. If there was a difference in systolic BP > 5 mmHg, a fourth measurement was taken and included in the average.

#### 2.6.2 Cardiorespiratory fitness

Cardiorespiratory fitness was assessed with a ramp ${\dot{V}}_{O_{2}\mathrm{peak}}$ test on a cycle ergometer (Lode Corival/Kettler Ergorace). Participants wore a face mask connected to a mixing chamber and metabolic cart (Quark CPET; COSMED; Chicago, IL, USA) for the measurement of gas exchange, as well as an HR monitor (H10 Sensor, Polar, Kempele, Finland) for the measurement of cardiovascular responses. Participants were instructed to maintain a pedalling cadence between 70 and 90 rpm throughout the test. The cycling protocol consisted of a 3‐min warm‐up at 50 W followed by progressively increased workload at a rate of 5 W every 10 s until volitional exhaustion. ${\dot{V}}_{O_{2}\mathrm{peak}}$ was deemed to have been achieved if a plateau in ${\dot{V}}_{O_{2}}$ was demonstrated in the final 30 s of the test or if two out of the following three criteria were reached: (1) rating of perceived exertion ≥17, (2) respiratory exchange ratio ≥1.13, or (3) heart rate ≥93% of age‐predicted maximum ([208–0.7 × age] × 0.93) (Wagner et al., 2020). ${\dot{V}}_{O_{2}\mathrm{peak}}$ was calculated as the average of the three highest 10‐s bins and expressed in both absolute and relative terms.

#### 2.6.3 Cognitive tests

Cognitive tests were administered via computerised testing software (Inquisit Lab Version 6, Millisecond, Seattle, WA, USA) on a laptop computer. All participants performed a practice round of the assessment battery in a familiarisation visit prior to the start of the study. The battery of cognitive tests included the digit symbol substitution task (DSST) and the Stroop test. The DSST was used to assess attention, processing speed and visuospatial memory (Jaeger, 2018). A nine‐symbol key was provided at the top of the page throughout the test, where symbols were matched to numbers in the range of one to nine. A grid was then presented with the symbols in random order and the participant was asked to press the number key associated with each symbol as quickly and accurately as possible for 120 s.

The Stroop test was used to assess inhibitory control and selective attention (Scarpina & Tagini, 2017). Participants were asked to identify the colour of text written on a screen using buttons describing four different colours. The text is presented in conditions that are considered congruent, incongruent or neutral. In the congruent condition, the word matches the colour of the text (e.g., ‘red’ written in red); in the incongruent condition, the word does not match the colour of the text (e.g., ‘red’ written in green); and in the neutral condition, the word is unrelated to the colour of the text (e.g., ‘dog’ written in blue). The primary outcome is Stroop cost, calculated as the difference in reaction time between the incongruent and congruent conditions.

#### 2.6.4 Blood processing and biochemical analysis

Venous blood samples were collected in EDTA tubes and centrifuged immediately at 3488 g at 4°C for 15 min. The resultant supernatant was aliquoted and stored at −80°C. A commercially available enzyme‐linked immunoassay kit (BEK‐2211‐2P, Biosensis, Thebarton, SA, Australia) was used to quantify plasma BDNF, which represents the biologically available pool of circulating BDNF. The intra‐ and inter‐assay coefficients of variation for this kit are 1.0% and 5.0%, respectively (Polacchini et al., 2015). All steps were followed in accordance with the manufacturer's instructions.

### 2.7 Statistical analysis

As this study presents a secondary outcome, the sample size calculation was based on vascular function (Cheng et al., 2025). All data and residuals were checked for normality using the Shapiro–Wilk test and inspection of the Q‐Q plots, and the Cook–Weisberg test, respectively. If data were not normally distributed, values were log‐transformed. For ${\dot{V}}_{O_{2}\mathrm{peak}}$, resting cardiovascular haemodynamics and physiological responses to the acute stimuli, one‐way ANOVAs were conducted because the number of time points was not consistent across all groups (i.e., HEATEX had 4 time points due to the combination of both heating and exercise). For cognitive function, linear mixed models were used to test for interactions between time (two levels: pre‐ and post‐intervention) and training group (four levels: CON, EX, HEAT, HEATEX) with time being a fixed factor and subjects as a random factor. When a significant main effect or interaction was found, pairwise comparisons were assessed using a Student's *t‐*test with a *post hoc* Bonferroni correction. The level of significance was set a priori at *P *< 0.05 and statistics were performed with StataSE Version 17.0 (StataCorp, College Station, TX, USA) or SPSS Version 21 (IBM Corp., Armonk, NY, USA). Figures were created with GraphPad Prism version 9 (GraphPad Software, Boston, MA, USA). All data are presented as means [SD] and include all participants except for one extreme outlier for Stroop cost that was identified by an iterative Grubbs's test (*P *< 0.05).

## 3 RESULTS

### 3.1 Participant characteristics

Sixty participants were randomly allocated into one of CON (*n* = 15), HEAT (*n* = 15), EX (*n* = 14) or HEATEX (*n* = 16). Participant characteristics are summarised in Table 1. On average, the cohort was young (age: 23 [3] years old), healthy (BMI: 24.0 [3.6] kg/m^2^; BP: 112 [9]/63 [6] mmHg) and moderately fit (${\dot{V}}_{O_{2}\mathrm{peak}}$: 42.5 [8.7] mL O_2_/kg/min).

**TABLE 1:** Participant characteristics.

	All (*n* = 60)	CON (*n* = 15)	HEAT (*n* = 15)	EX (*n* = 14)	HEATEX (*n* = 16)
Sex and gender, F and W (*n* (%))	30 (50%)	7 (47%)	8 (53%)	7 (50%)	8 (50%)
Age (years)	23 (3)	24 (4)	23 (3)	22 (4)	22 (2)
Height (m)	1.7 (0.1)	1.7 (0.1)	1.7 (0.1)	1.7 (0.1)	1.7 (0.1)
Weight (kg)	70.8 (14.3)	68.0 (12.3)	71.2 (13.7)	71.2 (17.1)	72.4 (15.0)
BMI (kg/m^2^)	24.0 (3.6)	23.2 (3.4)	24.4 (3.2)	24.9 (5.0)	23.6 (2.9)
Resting SBP (mmHg)	112 (9)	110 (9)	109 (10)	113 (7)	115 (9)
Resting DBP (mmHg)	63 (6)	64 (5)	63 (8)	63 (4)	63 (6)
Resting MAP (mmHg)	81 (6)	81 (6)	80 (8)	81 (4)	82 (7)
Resting HR (bpm)	63 (10)	64 (8)	61 (8)	64 (12)	64 (13)
${\dot{V}}_{O_{2}\mathrm{peak}}$ (mL/kg/min)	42.5 (8.7)	40.7 (8.7)	42.8 (6.1)	42.7 (11.7)	43.7 (8.2)

Data are means (SD). Abbreviations: BMI, body mass index; DBP, diastolic blood pressure; F, female; HR, heart rate; MAP, mean arterial pressure; SBP, systolic blood pressure; ${\dot{V}}_{O_{2}\mathrm{peak}}$, peak oxygen uptake; W, women.

### 3.2 Characterisation of intervention stimuli

The acute cardiovascular responses to each intervention session are outlined in Table 2 . Core temperature (*T*_core_) increased [HEAT, mean: 37.1 (CI: 36.9–37.3) to 37.5 (37.3–37.7)°C; EX, mean 37.2 (CI: 37.0–37.4) to 37.9 (37.7–38.1)°C; HEATEX, median: 37.1 (IQR: 0.4) to 37.8 (0.2)°C] in all groups (all *P *< 0.01) but CON (*P *= 0.29). Mean arterial pressure (MAP) increased in response to exercise only during EX and HEATEX (both *P *< 0.001), but heart rate increased in all groups (all *P *< 0.001) except CON (*P *= 0.28). Mean HR during all HEAT, EX and HEATEX training sessions was equivalent to 47%, 71% and 60% of HR_max_, respectively. Mean SR increased [HEAT, median: 142 (IQR: 98) to 465 (410) s^−1^; EX, median: 174 (IQR: 93) to 390 (309) s^−1^; HEATEX, median: 169 (IQR: 187) to 420 (233) s^−1^] in all groups (all *P *< 0.001) but CON (*P *= 0.52).

**TABLE 2:** Characterisation of intervention stimuli.

	CON		*P*	HEAT		*P*	EX		*P*	HEATEX		*P*
	*M*	95% CI/IQR		*M*	95% CI/IQR		*M*	95% CI/IQR		*M*	95% CI/IQR	
MAP (mmHg)			0.17			0.054			**<0.001**			**<0.001**
Pre	81	78, 85		80	77, 84		81 ^a^	77, 85		83 ^a^	9	
During (45)	86	82, 89		87	83, 90		97 ^b^	92, 101		104 ^b^	16	
During (90)										87 ^a^	9	
Post	84	80, 87		83	79, 87		83 ^a^	79, 87		84 ^a^	10	
HR (bpm)			0.28			**<0.001**			**<0.001**			**<0.001**
Pre	64	60, 68		59 ^a^	10		64 ^a^	58, 70		67 ^a^	20	
During (45)	67	62, 71		82 ^b^	21		134 ^b^	127, 140		140 ^b^	14	
During (90)										92 ^c^	15	
Post	62	58, 66		61 ^a^	16		71 ^a^	64, 77		70 ^a^	14	
*T*_core_ (°C)			0.29			**0.004**			**<0.001**			**<0.001**
Pre	37.0	36.8, 37.2		37.1 ^a^	36.9, 37.3		37.2 ^a^	37.0, 37.4		37.1 ^a^	0.4	
During (45)	36.9	36.7, 37.0		37.5 ^b^	37.3, 37.7		37.9 ^b^	37.7, 38.1		37.8 ^b^	0.2	
During (90)										37.6 ^b^	0.2	
Post	36.8	36.6, 37.0		37.3 ^a^	36.9, 37.3		37.6 ^ab^	37.3, 37.8		37.3 ^a^	0.3	
Mean SR (s^−1^)			0.52			**<0.001**			**<0.001**			**0.001**
Pre	129.5	96.7		142.2 ^a^	97.9		173.7 ^a^	92.9		169.0 ^a^	187.0	
During (45)	124.3	80.9		465.2 ^b^	410.1		389.5 ^b^	308.8		419.8 ^b^	232.6	
During (90)										373.6 ^b^	369.8	

Data are expressed as means (*M*) and 95% confidence intervals (CI) for parametric analyses and medians (*M*) and interquartile ranges (IQR) for nonparametric analyses. Outcomes were analysed using separate one‐way ANOVAs for each group with time as the independent variable. Significance indicators: *P*‐value shown in bold indicate a significant overall effect; all values with superscript “a” are not different from each other but the value with superscript “b” is different from all values with a superscript “a”. Abbreviations: DBP, diastolic blood pressure; HR, heart rate; MAP, mean arterial pressure; SBP, systolic blood pressure; SR, shear rate; *T*_core_, core temperature.

**TABLE 3:** Results from the digit symbol substitution task (DSST) completed in 120 s.

	Correct answers (*n*)	Errors (*n*)	Reaction time (s/correct answer)
CON			
Pre	56 (8)	1 (1)	2.2 (0.4)
Post	64 (10)	1 (1)	**1.9 (0.3)** ^*^
EX			
Pre	57 (9)	1 (1)	2.1 (0.4)
Post	61 (13)	1 (2)	2.1 (0.5)
HEAT			
Pre	65 (9)	0 (1)	1.9 (0.3)
Post	69 (14)	1 (1)	1.8 (0.3)
HEATEX			
Pre	61 (7)	2 (2)	2.0 (0.2)
Post	62 (8)	2 (2)	2.0 (0.2)
*P*			
Time	**<0.001**	0.771	**<0.001**
Group	0.089	**0.048**	0.111
Time × Group	0.119	0.563	**0.032**

Data are means (SD).

* Represents a difference between pre‐ and post‐intervention time points within a group determined by *P *< 0.05 with Bonferroni correction.

**TABLE 4:** Results from the Stroop cognitive test.

	Percentage correct incongruent	Percentage correct congruent	Reaction time incongruent (ms)	Reaction time congruent (ms)	Stroop cost (ms)
CON					
Pre	96 (3)	99 (2)	788 (102)	673 (59)	115 (93)
Post	97 (4)	99 (2)	724 (76)	643 (60)	81 (52)
EX					
Pre	96 (5)	98 (2)	790 (149)	702 (85)	88 (92)
Post	97 (4)	98 (3)	815 (108)	688 (86)	123 (103)
HEAT					
Pre	96 (5)	98 (3)	746 (73)	648 (64)	98 (58)
Post	96 (4)	98 (3)	706 (72)	635 (74)	71 (54)
HEATEX					
Pre	94 (5)	96 (4)	733 (84)	670 (56)	63 (72)
Post	95 (5)	98 (2)	726 (11)	665 (86)	61 (50)
*P*					
Time	0.232	0.694	0.222	0.060	0.581
Group	0.461	0.316	0.081	0.158	0.120
Time × Group	0.825	0.174	0.050	0.752	0.205

Data are mean (SD).

* Difference between pre‐ and post‐intervention time points within a group determined by *P *< 0.05 with Bonferroni correction.

### 3.3 Fitness, HR and BP changes with training

As reported previously in more detail (Cheng et al., 2025), there were no differences in the change in resting HR (*P *= 0.250), systolic BP (*P *= 0.410), diastolic BP (*P *= 0.500) or mean arterial pressure (*P *= 0.70) across intervention groups. There were no between‐group differences in absolute (*P *= 0.21) or relative ${\dot{V}}_{O_{2}\mathrm{peak}}$ (*P *= 0.55). The within‐group changes in aerobic capacity following the interventions were CON: mean absolute ${\dot{V}}_{O_{2}\mathrm{peak}}$: Δ0.06 [95% CI: −0.05, 0.17] L/min and mean relative ${\dot{V}}_{O_{2}\mathrm{peak}}$: Δ1.10 [95% CI: −0.48, 2.68] mL/kg/min; HEAT: mean absolute ${\dot{V}}_{O_{2}\mathrm{peak}}$: Δ0.18 [95% CI: 0.06, 0.29] L/min and mean relative ${\dot{V}}_{O_{2}\mathrm{peak}}$: Δ2.18 [95% CI: 0.60, 3.76] mL/kg/min; EX: mean absolute ${\dot{V}}_{O_{2}\mathrm{peak}}$: Δ0.11 [95% CI: −0.01, 0.22] L/min and mean relative ${\dot{V}}_{O_{2}\mathrm{peak}}$: Δ1.59 [95% CI: −0.05, 3.22] mL/kg/min; and HEATEX: mean absolute ${\dot{V}}_{O_{2}\mathrm{peak}}$: Δ0.21 [95% CI: 0.11, 0.32] L/min and mean relative ${\dot{V}}_{O_{2}\mathrm{peak}}$: Δ2.59 [95% CI: 1.06, 4.12] mL/kg/min.

### 3.4 Cognitive functions (Figure 1)

#### 3.4.1 Digit symbol substitution task (Table 3)

There was a main effect of time where the number of correct answers on the DSST increased from pre‐ to post‐intervention for all groups (*P *< 0.001). However, the were no group or interaction effects for the number of correct answers. There were no time or interaction effects for the number of errors, but there was a group effect where no *post hoc* differences were found between groups for the number of errors (all  *P* ≥ 0.378). There was no group effect for reaction time, but there were time and interaction effects where the CON group was only faster post‐intervention.

#### 3.4.2 Stroop test (Table 4)

There were no time, group or interaction effects on the Stroop test for the percentage of correct responses (congruent [*P *= 0.174] or incongruent [*P *= 0.825]), reaction time (congruent [*P *= 0.752] or incongruent [*P *= 0.050]), or Stroop cost (*P *= 0.205).

### 3.5 Plasma BDNF

There were no changes in plasma BDNF concentrations from baseline (CON: 647 [960] pg/mL; EX: 335 [399] pg/mL; HEAT: 1368 [2338] pg/mL; HEATEX: 859 [1362] pg/mL) to post‐intervention (CON: 583 [910] pg/mL; EX: 295 [331] pg/mL; HEAT: 1308 [1756] pg/mL; HEATEX: 837 [1381] pg/mL); however, these values were not normally distributed (Kolmogorov–Smirnov test: *P *< 0.001). Therefore, raw BDNF concentrations were log transformed (Kolmogorov–Smirnov test: *P *= 0.070) and are presented in Figure 2 where there were no significant main effects (group: *P *= 0.748, time: *P *= 0.189) or interactions (*P *= 0.417).

## 4 DISCUSSION

The purpose of this trial was to investigate the effects of 8 weeks of either exercise training, or local heat therapy or a combination of both on cognitive function and circulating BDNF. The main findings were that cognitive performance was not improved and there was no change in plasma BDNF following 8 weeks of aerobic exercise training or lower limb hot water immersion, alone or in combination, compared to a control group. These findings are in contrast with our hypothesis that post‐exercise local heating creates a larger physiological stimulus for improving cognitive performance and BDNF. Although the number of correct responses on the DSST was improved over time, this was not different between groups and is therefore consistent with practice effects typically observed with repeated testing (Walsh et al., 2020). Surprisingly, we also did not observe improved cognitive function in the exercise group, which perhaps indicates that the intervention stimuli were insufficient to induce cognitive changes in our young, healthy cohorts.

**FIGURE 1:**
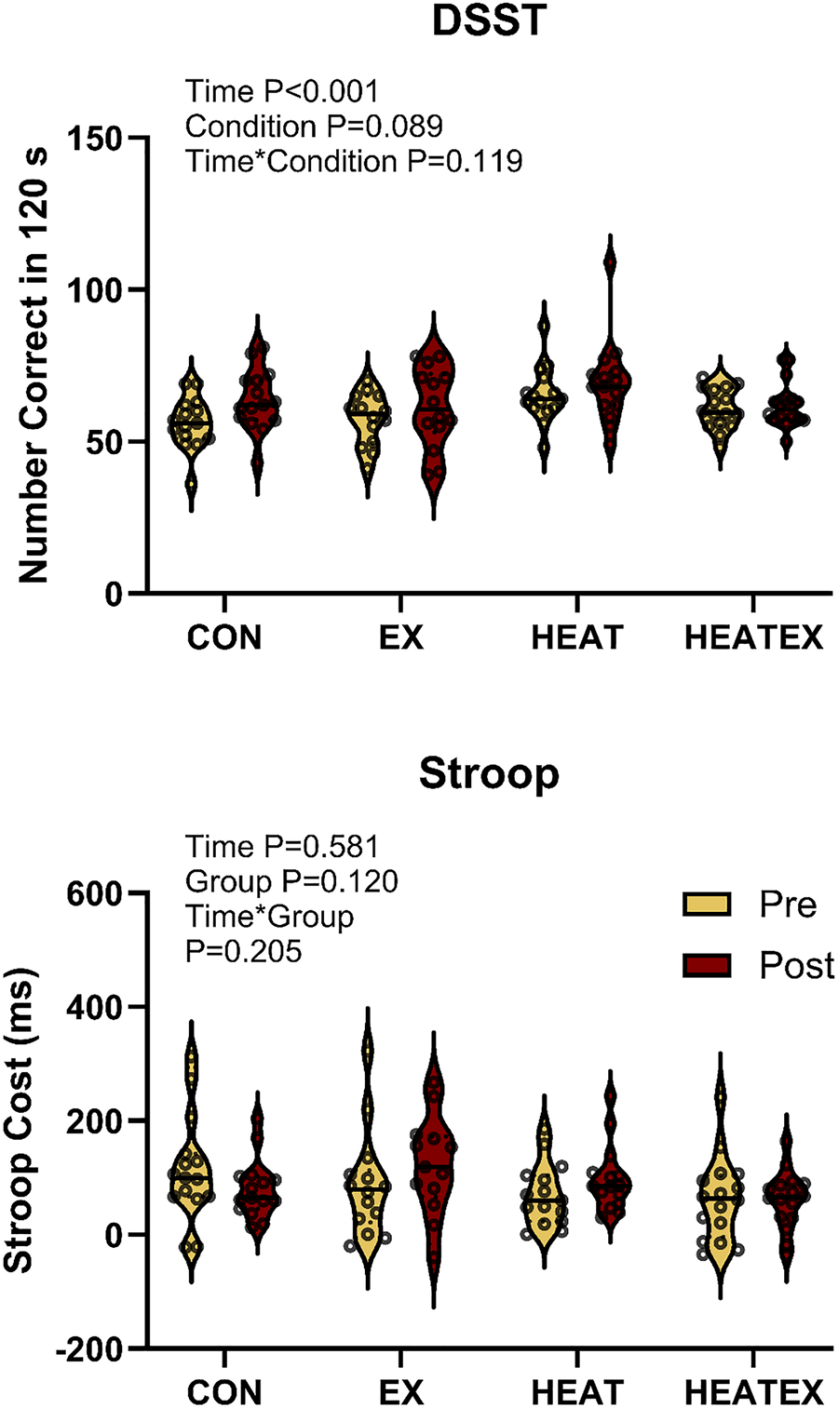
Number of correct responses in 120 s during the DSST (top) and the Stroop cost in time (incongruent–congruent) (bottom). Data are median (lines) and quartiles (violin plots).

To our knowledge, our study is the first to report the effects of longer‐term (8 weeks) local heating on cognitive outcomes. Schlader et al. (2015) tested whether acute passive heating by +1°C core temperature impacts cognitive performance in young and older adults. They reported that reaction time improved on tasks related to attentional and executive function domains following acute heat stress in both younger and older adults (Schlader et al., 2015). Barry et al. (2022) also tested cognition in response to a seven‐day passive heat acclimation protocol and found no changes to Stroop test performance with short‐term passive heating. Similar to acute heating (Schlader et al., 2015), we observed a main effect of the 8‐week interventions on DSST correct responses (attention task) in young, healthy adults, but this effect did not differ between groups (Figure 1). Further, there were no changes to reaction time or accuracy during the Stroop test (executive function) associated with the interventions.

**FIGURE 2:**
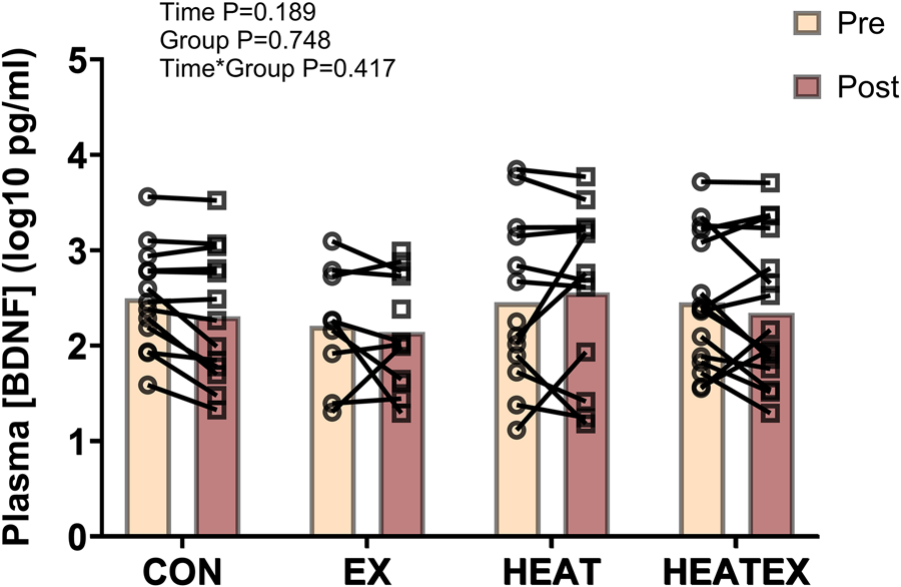
Concentrations of plasma BDNF before and after the 8‐week interventions of control (CON, pre *n* = 13, post *n* = 13), aerobic exercise (EX, pre *n* = 9, post *n* = 12), ankle heating (HEAT, pre *n* = 13, post *n* = 12), and combined exercise and heating (HEATEX, pre *n* = 16, post *n* = 16). Bars represent group means.

The mechanisms underlying the acute effects of heat exposure on cognition are likely multifactorial, and acute elevations in BDNF may contribute. Indeed, acute passive heating increases circulating BDNF in younger (Kirby et al., 2025; Ogawa et al., 2021) and older (Kirby et al., 2025) adults. Interestingly, Ogawa et al. found that 90 min of whole‐body heating (+1.8°C core temperature) significantly increased circulating BDNF, whereas 90 min of localised heating of the legs (+0.4°C) had no effect on BDNF compared to a control condition (Ogawa et al., 2021). However, this study also employed upper body cooling during leg heating, which decreased brachial blood flow by ∼50% (Ogawa et al., 2021). This latter point is important when compared to our study, where brachial artery shear rate increased by 327% (Table 2) despite a similar rise in core temperature during local heating (+0.4°C). Accordingly, localised heating, such as the protocol used in the current study, may be an insufficient stimulus to increase circulating BDNF. Indeed, in the current study, there was no change in the concentration of plasma BDNF (Figure 2), which is possibly due to the low systemic perturbation associated with the lower limb thermal stress (change in core temperature +0.4°C) (Cheng et al., 2025). However, measurements of serum BDNF as well are required to confirm this possibility, given that Ogawa et al. reported increased serum, but not plasma, BDNF with lower body heating (Ogawa et al., 2021). It would be expected that the increase in vascular shear stress in the legs as a result of cycling exercise or hot water immersion would stimulate the release of BDNF from endothelial cells (Dinoff et al., 2017). Perhaps it is the case that a larger endothelial cell activation (e.g., whole‐body) or greater physiological stimulus (e.g., higher exercise intensity) is required to release sufficient BDNF to increase circulating concentrations.

The benefits of exercise for brain health and the prevention of cognitive decline are well documented (Basso & Suzuki, 2016; Fernandes et al., 2017). For example, there is a cross‐sectional association between higher aerobic fitness and better cognition across the lifespan (Voss et al., 2011). Moreover, a meta‐analysis reported that exercise causes acute improvements to cognitive function, specifically in the domain of executive functions (Verburgh et al., 2014). These acute benefits of exercise may represent a ‘window’ of cognitive priming after which exercise causes a temporary improvement in cognitive functions, potentially due to elevated arousal measured via electroencephalography (Kamijo et al., 2007). Exercise may also facilitate the consolidation of memory, although this effect may be delayed compared to the immediate effects of exercise on executive functions (van Dongen et al., 2016). These effects repeated over time may cause long‐term changes in brain structure and function (Gomez‐Pinilla & Hillman, 2013). For example, structural brain changes occur with exercise training, but a biochemical stimulus is required for such neuroplastic adaptations (Mandolesi et al., 2018). A key mechanism by which exercise may promote cognitive improvements is through increased circulating concentrations of BDNF, which is a putative neurochemical that mediates neurogenesis in response to exercise training (Intlekofer et al., 2013). Meta‐analysis data show that acute aerobic exercise is a robust stimulus that results in increased plasma BDNF concentrations – a result that is partially associated with both exercise duration and intensity (Dinoff et al., 2017). Indeed, acute aerobic exercise for approximately 40 min at ∼60% ${\dot{V}}_{O_{2}\mathrm{peak}}$ is linked to a 60% increase in plasma BDNF (Dinoff et al., 2017), which is similar to the exercise used in our study (∼70% HR_max_). Similarly, acute sessions of passive heating (>1.5°C increase in core temperature) appear to elicit increased *plasma* BDNF concentrations as well (Kojima et al., 2018; Ogawa et al., 2021), whereas lower‐body heating (+0.4°C) may increase *serum* BDNF, which is similar to the change in core temperature in this study.

When considering the chronic effects of exercise, a separate meta‐analysis reported that resting concentrations of BDNF are elevated following aerobic, but not resistance, exercise training (Dinoff et al., 2016). However, no studies have measured BDNF following longer‐term passive heating. The effects of chronic exercise training are not as well understood in young and healthy adults, but evidence indicates that cognition is improved in the visuospatial memory domain (Stroth et al., 2009). The cognitive benefits of exercise interventions are also highlighted by individual variability, where larger improvements have been reported with greater changes in fitness across the intervention, potentially mediated by larger increases in BDNF concentrations (Heisz et al., 2017). Our study partially aligns with this effect, where reaction time was improved over the intervention period, but this was not specific to exercise. Importantly, our study reports no effect of the intervention period on executive functioning regardless of condition. Thus, it is perhaps possible that exercise causes a short‐term potentiation of cognition rather than chronic changes to baseline status; however, this is a speculative contention that is subject to various stipulations discussed in the limitations section below (e.g., lack of improvement in fitness).

### 4.1 Perspectives

With advancing age, dementias become very prevalent, with upwards of 5–10% of the population 65+ diagnosed with some form, a figure which doubles every 4–5 years (Gorelick et al., 2011). Although Alzheimer's is the most commonly diagnosed type of dementia, the majority of cases are in fact mixed dementias with some form of vascular pathology (Skrobot et al., 2018). The effects of physical activity on cardiovascular risk factors and changes in cognitive function have been reported (Larson et al., 2006; Pedersen & Saltin, 2015); however, physical activity rates remain poor amongst the general population for various reasons, including lack of time, interest or physical ability (Colley et al., 2011). Thus, alternative approaches to maintain and improve brain health are warranted. Not only can whole‐body passive heating reduce cardiovascular risk factors (Brunt et al., 2016), but epidemiological data also link sauna use to reduced risk of stroke and Alzheimer's (Kunutsor et al., 2018; Laukkanen et al., 2017). The underlying mechanisms may be underpinned by molecular factors (such as heat shock proteins, BDNF) associated with heating, which may have many systemic and cerebral benefits (Brunt & Minson, 2021; Brunt et al., 2018; Coombs & Tremblay, 2019). Post‐exercise passive heating also prolongs the physiological stimulus of exercise and may be a way to extend ‘exercise’ duration in those with limited physical capacity (older adults, diseases) (Zurawlew et al., 2019). Although the appeal of the foot spa/bath is its affordability, accessibility and ease of use, given its ineffectiveness in this study, a potential solution may be to increase the public's accessibility to modes of heat therapy that have a larger evidence base. For example, this may come in the form of installing hot tubs or saunas in free/low‐cost community centres, gyms, etc.

### 4.2 Limitations

There are a few qualifications to acknowledge about the present study that pertain to limitations in our ability to generalise the findings, given the choices made in the study population and design, which were made to provide real‐world application. The first is the study population, which consists of young, healthy people, primarily university students. Although previous studies have reported improvements to cognition in young and healthy adults, this may depend on baseline status (Voss et al., 2011). Indeed, the participants in this study were of relatively high fitness (Table 1) and were highly educated, which may indicate a ceiling effect of exercise and education on the chosen cognitive tests. Additionally, given the relatively high aerobic fitness of the groups at baseline, the aerobic exercise training programme was not associated with improvements in ${\dot{V}}_{O_{2}\mathrm{peak}}$ despite a mid‐study peak test to facilitate accurate training progression. This finding indicates that a larger training stimulus was likely required to improve fitness, and thus may also be necessary to improve cognition. Finally, we only measured plasma BDNF in the current study, but it is possible that changes to serum BDNF might be more detectable, particularly during lower‐body heating (Ogawa et al., 2021).

Given that access to various methods of whole‐body passive heating (e.g., hot tub, sauna, etc.) may be limited amongst the general population, lower limb hot water immersion was chosen for the current study as an inexpensive, portable and accessible option. Studies have reported improvements to local vascular function with several weeks of lower limb (Carter et al., 2014b) or forearm heating (Carter et al., 2014a); however, studies reporting systemic and/or cellular improvements in response to passive heating have used whole‐body hot water immersion (Brunt et al., 2018). Accordingly, it is possible that greater thermal strain was required for observable improvements to the cardiovascular/systemic systems that could extend to cognitive benefits. Indeed, Ogawa et al. (2021) reported greater concentrations after acute whole‐body heating where core temperature increased by >1°C, whereas there was a smaller change to core temperature with the lower limb heating protocol used in the current study (Cheng et al., 2025).

### 4.3 Conclusion

This study demonstrates that the independent effects of exercise and lower limb local heating on the cardiovascular system and brain do not combine in an interactive manner to improve cognition or circulating concentrations of BDNF after an 8‐week intervention in young, healthy adults. Future research should consider using a greater thermal stimulus and/or a study population with lower cognitive or cardiorespiratory function at baseline.

## AUTHOR CONTRIBUTIONS

Conception or design of the work: Jem L. Cheng, Maureen J. MacDonald and Jeremy J. Walsh. Acquisition, analysis or interpretation of data for the work: Jem L. Cheng, Geoff B. Coombs, Christina Pizzola, Keira Mattook, Calvin Armstrong, Maureen J. MacDonald and Jeremy J. Walsh. Drafting the work or revising it critically for important intellectual content: Jem L. Cheng, Geoff B. Coombs, Christina Pizzola, Keira Mattook, Calvin Armstrong, Maureen J. MacDonald and Jeremy J. Walsh. All authors have read and approved the final version of this manuscript and agree to be accountable for all aspects of the work in ensuring that questions related to the accuracy or integrity of any part of the work are appropriately investigated and resolved. All persons designated as authors qualify for authorship, and all those who qualify for authorship are listed.

## CONFLICT OF INTEREST

There are no conflicts of interest, financial or otherwise.

## Data Availability

The data that support the findings of this study are available from the corresponding author upon reasonable request.
